# Supplementary material for: Assessing the reliability and validity of the Slovenian version of the Appraisal of Diabetes Scale (ADS-S) in type 2 diabetes patients
Source: PLoS One. 2024 Mar 25;19(3):e0300797. doi: 10.1371/journal.pone.0300797 (PMC10962803; doi:10.1371/journal.pone.0300797)
Supplement: S1 Appendix — (DOCX) [file pone.0300797.s001.docx]

**S1 Appendix: English version of the Appraisal of Diabetes Scale (ADS)**

People differ in their thoughts and feelings about having diabetes. We would like to know how you feel about having diabetes. Therefore, please circle the answer to each question which is closest to the way you feel. Please give you honest feelings – we are interested in how you feel, not what your doctor or family may think.

1. How upsetting is having diabetes for you?

| 1  Not at all | 2  Slightly upsetting | 3  Moderately upsetting | 4  Very upsetting | 5  Extremely upsetting |
| --- | --- | --- | --- | --- |

1. How much control over your diabetes do you have?

| 1  None at all | 2  Slight amount | 3  Moderate amount | 4  Large amount | 5  Total amount |
| --- | --- | --- | --- | --- |

1. How much uncertainty do you currently experience in your life as a result of being diabetic?

| 1  None at all | 2  Slight amount | 3  Moderate amount | 4  Large amount | 5  Extremely large amount |
| --- | --- | --- | --- | --- |

1. How likely is your diabetes to worsen over the next several years? (Try to give an estimate based on your personal feeling rather than based on a rational judgement.)

| 1  Not likely at all | 2  Slightly likely | 3  Moderately likely | 4  Very likely | 5  Extremely likely |
| --- | --- | --- | --- | --- |

1. Do you believe that achieving good diabetic control is due to your efforts as compared to factors which are beyond your control?

| 1  Totally because of me | 2  Mostly because of me | 3  Partly because of me and partly because of other factors | 4  Mostly because of other factors | 5  Totally because of other factors |
| --- | --- | --- | --- | --- |

1. How effective are you in coping with your diabetes?

| 1  Not at all | 2  Slightly effective | 3  Moderately effective | 4  Very effective | 5  Extremely effective |
| --- | --- | --- | --- | --- |

1. To what degree does your diabetes get in the way of your developing life goals?

| 1  Not at all | 2  Slight amount | 3  Moderate amount | 4  Large amount | 5  Extremely large amount |
| --- | --- | --- | --- | --- |

***Reference****: Carey MP, Jorgensen RS, Weinstock RS, Sprafkin RP, Lantinga LJ, Carnrike CL, et al. Reliability and validity of the appraisal of diabetes scale. J Behav Med. 1991;14(1):43-51. doi: 10.1007/BF00844767.*
