# Supplementary material for: Assessing the reliability and validity of the Slovenian version of the Appraisal of Diabetes Scale (ADS-S) in type 2 diabetes patients
Source: PLoS One. 2024 Mar 25;19(3):e0300797. doi: 10.1371/journal.pone.0300797 (PMC10962803; doi:10.1371/journal.pone.0300797)
Supplement: S2 Appendix — (DOCX) [file pone.0300797.s002.docx]

**S2 Appendix: Slovenian version of the Appraisal of Diabetes Scale (ADS-S)**

Mišljenje in občutki ljudi glede lastne sladkorne bolezni se razlikujejo. Zanima nas, kako se zaradi sladkorne bolezni počutite vi, zato vas prosimo, da pri vsakem vprašanju obkrožite tisti odgovor, ki je najbližji vašim občutkom. Prosimo, da ste iskreni. Zanima nas, kako se počutite vi in ne, kaj meni vaš zdravnik ali vaša družina.

1. Kako moteča je za vas sladkorna bolezen?

| 1  Sploh ne | 2  Nekoliko moteča | 3  Zmerno moteča | 4  Zelo moteča | 5  Izjemno moteča |
| --- | --- | --- | --- | --- |

1. V kakšni meri imate nad svojo sladkorno boleznijo nadzor?

| 1  Sploh ne | 2  Nekoliko | 3  Zmerno | 4  Zelo | 5  Popolnoma |
| --- | --- | --- | --- | --- |

1. Koliko negotovosti doživljate v svojem življenju, ker ste sladkorni bolnik?

| 1  Sploh nič | 2  Nekoliko | 3  Zmerno | 4  Veliko | 5  Izjemno veliko |
| --- | --- | --- | --- | --- |

1. Kako verjetno je, da se bo vaša sladkorna bolezen poslabšala v naslednjih nekaj letih (poskušajte podati oceno na osnovi lastnih občutkov in ne na podlagi razumskega razmišljanja)?

| 1  Sploh ni  Verjetno | 2  Malo  verjetno | 3  Zmerno  verjetno | 4  Zelo  verjetno | 5  Izjemno  Verjetno |
| --- | --- | --- | --- | --- |

1. Ali menite, da je dober nadzor nad sladkorno boleznijo bolj odvisen od vašega truda kot od drugih dejavnikov, ki niso pod vašim nadzorom?

| 1  Popolnoma odvisen od mene | 2  Večinoma  odvisen od mene | 3  Delno odvisen od mene in delno od drugih dejavnikov | 4  Večinoma odvisen od drugih dejavnikov | 5  Popolnoma odvisen od drugih dejavnikov |
| --- | --- | --- | --- | --- |

1. Kako učinkoviti ste pri obvladovanju svoje sladkorne bolezni?

| 1  Sploh nisem | 2  Malo sem učinkovit | 3  Zmerno sem učinkovit | 4  Zelo sem učinkovit | 5  Izjemno sem učinkovit |
| --- | --- | --- | --- | --- |

1. V kakšni meri vas sladkorna bolezen ovira pri razvijanju življenjskih ciljev?

| 1  Sploh me ne | 2  V majhni meri | 3  Zmerno | 4  V veliki meri | 5  Izjemno |
| --- | --- | --- | --- | --- |
